# Ergebnisse des Limberg-Plastik-Verfahrens bei akuten und chronischen Pilonidalabszessen

**DOI:** 10.1007/s00104-021-01439-0

**Published:** 2021-06-16

**Authors:** Jamal Driouch, C. Braumann, J. Dehnst, M. Ikram, G. Alnammous, D. Bausch, T. Glatz

**Affiliations:** 1 Chirurgische Klinik – Allgemein- und Viszeralchirurgie, Gefäßchirurgie, Marien Hospital Herne – Universitätsklinikum, Ruhr-Universität Bochum, Hölkeskampring 40, 44625 Herne, Deutschland; 2 Klinik für Chirurgie, St. Josef Hospital Bochum, Ruhr-Universität Bochum, Gudrunstraße 56, 44791 Bochum, Deutschland; 3 Klinik für Chirurgie, Paracelsus-Klinik Hemer, Breddestraße 22, 58675 Hemer, Deutschland

**Keywords:** Sinus pilonidalis, Sakrokokzygeale Fistel, Sakrokokzygealer Abszess, Komplikationen, Hautlappen, Pilonidal sinus, Sacrococcygeal fistula, Sacrococcygeal abscess, Complications, Skin flap

## Abstract

**Hintergrund:**

In der Behandlung des Sinus pilonidalis werden unterschiedliche Therapiealgorithmen für den akuten sowie den chronischen Sinus pilonidalis empfohlen. Während sich beim chronischen Sinus pilonidalis ein einzeitiges Vorgehen als Exzision oder plastische Rekonstruktion nach Limberg oder Karydakis anbietet, ist die empfohlene Vorgehensweise beim akuten Pilonidalabszess zweizeitig. Ziel dieser Studie war es, die Ergebnisse der einzeitigen Operation mit Limberg-Plastik bei akutem Pilonidalabszess und chronischem Sinus pilonidalis bezogen auf Rezidive, Wundheilungsstörungen, stationärer Liegedauer sowie Patientenzufriedenheit zu vergleichen.

**Methoden:**

Von 2009 bis 2014 wurden 39 Patienten in die prospektive Beobachtungsstudie eingeschlossen. 21 Patienten mit akutem Pilonidalabszess, 18 mit chronischem Sinus pilonidalis. Alle Patienten wurden einzeitig mittels Limberg-Rautenplastik operativ behandelt. Die Gruppen wurden in Bezug auf postoperative Komplikationsrate und Rezidivhäufigkeit miteinander verglichen.

**Ergebnisse:**

Beide Gruppen waren im Wesentlichen vergleichbar. Die Analyse der postoperativen Ergebnisse zeigte eine vergleichbare Rate an Wundheilungsstörungen (10 % vs. 17 %, *p* = 0,647). In der Gruppe des akut abszedierten Sinus trat kein Rezidiv im Beobachtungszeitraum auf, während sich in der chronischen Gruppe 2 (11 %) Rezidive zeigten (*p* = 0,206).

**Diskussion:**

Die Ergebnisse der Limberg-Plastik als einzeitige Therapie des Pilonidalabszesses sind mit denen beim chronischen Sinus pilonidalis vergleichbar. Es zeigt sich ein Trend zu einem geringeren Rezidivrisiko. Der Einsatz der Limberg-Plastik scheint daher auch in der akuten Infektsituation eine adäquate Therapieoption.

## Abstract (en)

**Background:**

A number of different treatment algorithms are recommended for the treatment of an acute pilonidal abscess and a chronic pilonidal sinus. While a 1-stage surgical procedure using excision or plastic reconstruction according to Limberg or Karydakis is suggested for chronic pilonidal sinus, a 2‑stage procedure is recommended for an acute pilonidal abscess. The aim of this study was to compare the results of the 1‑stage surgery with plastic reconstruction according to Limberg for acute pilonidal abscess and chronic pilonidal sinus in terms of recurrence, disorders of wound healing, inpatient length of stay and patient satisfaction.

**Methods:**

From 2009 to 2014 a total of 39 patients were included in this prospective observational study: 21 patients with acute pilonidal abscess and 18 patients with chronic pilonidal sinus. All patients were surgically treated with a 1‑stage procedure using the Limberg flap method. The groups were compared in terms of postoperative complication rates and frequency of recurrence.

**Results:**

Both groups were basically comparable with respect to demographic characteristics and risk factor profiles. Analysis of the postoperative results showed a comparable rate of postoperative wound healing disorders (10% vs. 17%, *p* = 0.647). In the group with acute pilonidal abscesses there was no recurrence during the observational period, while in the chronic pilonidal sinus group there were 2 (11%) recurrences (*p* = 0.206).

**Conclusion:**

The results of the Limberg flap procedure regarding acute pilonidal abscesses were comparable to those of chronic pilonidal sinus. The results of this study show a trend to a lower risk of recurrence. The use of the Limberg flaps therefore also seems to be an adequate treatment option in an acute infection situation.

Der akut oder chronisch entzündete Sinus pilonidalis stellt für den Patienten ein schmerzhaftes und störendes Krankheitsbild dar. Die herkömmliche Therapie im Sinne einer offenen Exzision ist mit einer langwierigen offenen Wundheilung und narbigen Abheilung verbunden [1, 2]. Die einzeitige Exzision mit plastischer Deckung im Sinne einer Limberg-Plastik in gleicher Sitzung ist geeignet, den Wundheilungsprozess zu beschleunigen, ist aber mit einer erhöhten Rate an postoperativen Komplikationen und Rezidiveingriffen verbunden. Insbesondere bei akut-abszedierten Befunden wird dieses Operationsverfahren daher kritisch gesehen und nur sporadisch eingesetzt.

## Hintergrund

Der Sinus pilonidalis stellt eine entzündliche Reaktion der Haut und des subkutanen Fettgewebes dar, die eine akute sowie eine chronische Verlaufsform haben kann und häufig junge Männer zwischen dem 20. Und 30. Lebensjahr betrifft [3]. Hodges leitete 1880 den Namen aus dem Lateinischen pilonidal von „pilus“ (Haar) und „nidus“ (Nest) ab [4]. Der Pilonidalsinus ist am Oberrand der Rima ani, oberhalb des Steißbeins lokalisiert. Anders als in der Vergangenheit wird nicht von einer genetischen Disposition als Ursache ausgegangen, sondern als erworbene Folge von wurzelnah abgebrochenen Haaren, die in die Tiefe eindringen und eine Fremdkörperreaktion auslösen [5]. Als Prädispositionsfaktoren werden in den deutschen S3-Leitlinien eine starke Behaarung sowie übermäßige Schweißbildung angegeben [6, 7]. Auch eine unzureichende Körperhygiene, Adipositas und eine damit assoziierte tiefe Analfalte sowie sitzende Tätigkeiten werden als begünstigende Faktoren diskutiert.

Für die operative Behandlung des chronischen Sinus pilonidalis sind verschiedene Operationsverfahren verfügbar, als etabliertes Verfahren die radikale Exzision und Einleitung einer sekundären Wundheilung. Dieses Verfahren aber geht mit einer langen Wundheilungsdauer und längerem Arbeitsausfall einher. Auch die Rezidivrate ist nicht unerheblich. Minimal-invasive Techniken wie Pit-Picking, Lay-open (Fistelspaltung), endoskopische Methoden sowie Laserbehandlung stellen ebenfalls aktuelle Therapiemethoden dar.

Mit der Exzision und plastischen Deckung des Defekts in einer Sitzung sollen Kriterien wie langwierige Wundheilungen und hohe Rezidivraten verhindert werden [8–11]. Auch sollte die stationäre Liegedauer kurz und der Arbeitsausfall niedrig gehalten werden [12]. Die Limberg-Rautenplastik ist zwar nicht einfach durchführbar, erfüllt jedoch sämtliche der erwähnten Kriterien. Daher wird die plastische Rekonstruktion nach Limberg sowie Karydakis in der Behandlung des Sinus pilonidalis zunehmend angewandt [3, 4, 6, 7, 13, 14]**.**

Als Operationsverfahren des Sinus pilonidalis stellt sie, neben der Karydakis-Plastik, die am häufigsten in Deutschland angewandte plastische Operationsmethode dar [6, 7]. Mit dem Abflachen der Analfalte sollen Reibeeffekte der Pofalte und das Eindringen der abgebrochenen Haare in die Haut vermieden werden. Mit einer kurzen Rekonvaleszenzdauer und zügigen Wiederaufnahme der Arbeit stellt sie eine gute operative Alternative für den Patienten dar.

Die aktuelle S3-Leitlinie 2020 empfiehlt zur Versorgung eines akut abszedierenden Sinus pilonidalis die primäre Inzision und in zweiter Sitzung die definitive Versorgung mittels Schwenklappenplastik. Es wird auf kleinere Inzisionen gegenüber radikaleren als definitive Resektion hingewiesen. Beim chronischen Sinus pilonidalis wird bei der radikalen Exzision mit offener Wundbehandlung die Empfehlung ausgesprochen, primär kleinere Exzisionen durchzuführen [6].

Die offene Wundbehandlung mit sekundärer Wundheilung stellt ein einfaches und sicheres Verfahren dar, die Nachteile sind bereits erörtert worden. Bei der zweizeitigen Vorgehensweise muss sich der Patient zwei Operationen und somit zwei Narkosen unterziehen. Auch dies ist mit einem längeren Arbeitsausfall verbunden. Daher fordern einzelne Autoren eine definitive operative Sanierung eines akut abszedierenden Pilonidalsinus bereits in erster Sitzung [10, 11]. Dies erspart den betroffenen Patienten einen zweiten operativen Eingriff und verringert zudem berufliche Ausfallzeiten [13]. Die bestmögliche operative Versorgung des akut abszedierenden Sinus pilonidalis wird weiterhin kontrovers diskutiert [6, 7, 15].

Einerseits ist die Therapiezielsetzung der einzeitigen Limberg-Plastik wie beim chronischen Sinus pilonidalis die Aufhebung der Analfalte, zur Vermeidung der Haargrübchenbildung und somit Senkung des Rezidivrisikos, Patientenzufriedenheit und ästhetische Ergebnisse. Andererseits wird kritisch hinterfragt, ob ein akuter Pilonidalabszess in primärer Sitzung mittels Lappenplastik sicher versorgbar ist. Ein akuter Sinus pilonidalis lässt keinen Spielraum zur intraoperativen Fisteldarstellung mittels Blaufärbung, sodass die Detektion und Resektion kleiner Fistelgänge intraoperativ unsicher ist. Die Entzündung des umgebenden Weichteilgewebes könnte Anlass geben, den Defekt größer auszuschneiden, als dies im entzündungsfreien Intervall wäre. Ein erhöhtes Risiko für postoperative Wunddehiszenzen der ödematös geschwollenen Wundränder wäre anzunehmen. Auch bestünde die Gefahr der bakteriellen Transposition in die Wundumgebung [5, 6]. Ausgehend von der o. g. Kritik wird die Hypothese gestellt, dass chirurgische Komplikationen sowie Rezidive nach der Limberg-Plastik bei „akutem Pilonidalabszess“ häufiger zu erwarten sind als beim „chronischen Sinus pilonidalis“.

## Methodik

### Studiendesign

In diese prospektive Beobachtungsstudie wurden vom November 2009 bis Dezember 2014 39 Patienten eingeschlossen, die sich aufgrund eines Sinus pilonidalis in der chirurgischen Ambulanz des Krankenhauses Elsey vorstellten. Das hier beschriebene Operationsverfahren mittels einzeitiger Exzision und Rekonstruktion nach Limberg wurde insgesamt 58 Patienten, die die Einschlusskriterien der Studie erfüllten, sowohl mit akut entzündetem (*n* = 30) als auch chronischem (*n* = 28) Piloninalsinus angeboten und erläutert. Alle im Hintergrunddienst tätigen Operateure hatten Expertise in der Limberg-Technik. 21 aus der „akuten Gruppe“ und 18 aus der „chronischen Gruppe“ stimmten der einzeitigen Operation mit plastischer Rekonstruktion nach Limberg in gleicher Sitzung zu und wurden nach schriftlicher Einwilligung in die Studie eingeschlossen. 9 der 30 Patienten mit akutem Pilonidalsinus lehnten die Limberg-Plastik ab und wurden mittels kleiner Inzision unter systemischer Analgesie, Antibiotikatherapie und lokaler Vereisung ambulant oder mittels Exzision in Narkose und offener Wundheilung behandelt. 10 der 28 Patienten mit chronischem Sinus lehnten ebenfalls die Versorgung nach Limberg ab, es wurde eine komplette Exzision mit Teilverschluss oder komplett offener Wundheilung durchgeführt.

Die Zahl der im Beobachtungszeitraum insgesamt behandelten Patienten lag mit 20 bis 25 Patienten pro Jahr deutlich höher, aus logistischen Gründen (Kapazitätsgründe, Ausschlusskriterien der Studie sowie zu Beginn des Studienzeitraums fehlende Vigilanz) wurde das Studienverfahren nur etwa der Hälfte der Patienten angeboten, eine systematische Selektion nach Risikofaktoren oder Operateursneigung erfolgte nicht. So wurden auch adipöse Patienten mit Begleiterkrankungen und großen Abszessen eingeschlossen, eine gewisse Selektion des Patientengutes lässt sich jedoch retrospektiv nicht sicher ausschließen, da eine vollständige Erfassung aller Patienten mit dem genannten Krankheitsbild nicht erfolgt ist.

Als Ausschlusskriterien galten:

- Rezidive nach vorausgegangenen plastischen Operationen,
- In-sano-Exzisionen,
- große Defekte, die kein Material für eine Schwenklappenplastik übrigließen.

Einschlusskriterien waren:

- primärer Pilonidalabszess,
- chronischer Sinus pilonidalis,
- Rezidive nach ein- oder mehrmals lokalen Inzisionen,
- Rezidive nach Einleitung einer offenen Wundheilung.

Ein Abschluss der Wundheilung wurde als Bedingung für die Operationsindikation nach Limberg vorausgesetzt.

In dieser Studie erfolgte die Definition des akuten Pilonidalabszess durch die Entzündungskriterien Tumor, Rubor, Calor und Dolor. Der Abszess ist schmerzhaft, in einem kurzen Zeitintervall aufgetreten mit entzündlich geschwollener Umgebung und gerötet sowie überwärmt definiert. Eine spontane Perforation ist noch nicht eingetreten. Der chronische Sinus pilonidalis ist dadurch gekennzeichnet, dass mindestens ein Fistelostium mit rezidivierendem übelriechendem Sekretausfluss existiert.

Präoperativ wurden potenzielle Risikofaktoren für das Auftreten von postoperativen Wundheilungsstörungen und Rezidiven systematisch erfasst. Als Nikotinabusus wurde hier der anhaltende regelmäßige inhalative Nikotingenuss von kumulativ mindestens 5 „pack years“ definiert. Die postoperative Patientenzufriedenheit wurde zur einfachen Dokumentation mit subjektiver Zufriedenheit oder Unzufriedenheit angegeben.

### Operative Technik

**Figure 1:**
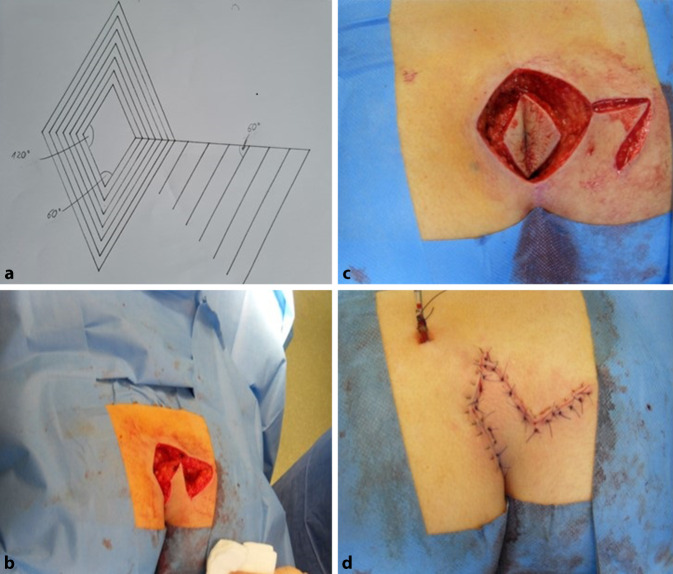

Als Landmarke wird eine vorgefertigte rautenförmige Schablone, in verschiedenen Größen, individuell an die Größe des Defektes angepasst, gewählt. Der Defekt wird in die rhomboide Schablone zentriert [16, 17]. Die Raute sollte vier gleich lange Seiten haben, zwei gegenüberliegende Winkel mit 60° und zwei gegenüberliegende Winkel mit 120°. Entlang der Landmarke nach vorherigem Anzeichnen erfolgt der rhomboide Hautschnitt, das subkutane Fettgewebe wird mit dem monopolaren Strom bis auf die Sakralfaszie inzidiert. Beim chronischen Sinus pilonidalis wird vorher die Blaufärbung durch das Fistelostium injiziert. Auf der Sakralfaszie wird das zu exzidierende infizierte Gewebe rautenförmig ausgeschnitten und zur histologischen Untersuchung abgegeben. Anschließend wird rechts des rhomboidförmigen Defektes gluteal ein dreieckiger Lappen gewählt, wobei der innere Schnitt einen Winkel von 120° haben sollte und der äußere Schnitt 60°, die Schenkel sollten parallel zur Raute verlaufen [15–17]. Der dreieckige Lappen wird dann mobilisiert und in den Defekt hineingeschwenkt (Abb. 1). Nach Einlage von 1 bis 2 14er-Redondrainagen, die lateral ausgeleitet werden, wird der Schwenklappen zweischichtig subkutan in Einzelknopftechnik adaptiert. Die Haut wird nach Donati in Einzelknopftechnik verschlossen.

### Prä- und postoperatives Management

Die Limberg-Plastik war mit einer stationären Behandlung von 5 bis 7 Tagen sowie einer antibiotischen Therapie mit Cefuroxim 1500 mg und Clont 500 mg verbunden, die über den gesamten stationären Aufenthalt 2‑mal täglich intravenös verabreicht wurde. Präoperativ erfolgte am Morgen des Operationstages eine lokale Rasur des Operationsfeldes bis 10 cm lateral der Rima ani, kranial zum Oberrand der Beckenschaufel sowie kaudal bis zum Anus. Eine Darmvorbereitung wurde als nicht notwendig eingestuft. Die Operation erfolgte in Bauchlage und Intubationsnarkose. Die perioperative Antibiotikatherapie wurde während der Narkoseeinleitung ungefähr 30 min vor Schnitt gegeben.

Es erfolgten täglich postoperative und alle 2 bis 3 Tage poststationäre Nachbehandlungen sowie Kontrollen bis zum Entfernen des Nahtmaterials am 15. postoperativen Tag. Komplikative Verläufe wurden häufiger und ggf. täglich nachgesorgt. Abschließend wurde eine telefonische Auskunft über die subjektive Zufriedenheit nach einem Jahr eingeholt. Die schriftliche Zustimmung der Patienten, ihrer Erziehungsberechtigten oder Betreuer lag vor.

### Statistische Auswertung

Die Auswertung der Daten erfolgte retrospektiv aus unserer prospektiv erhobenen Datenbank mittels SPSS© für Windows (Version 27; IBM SPSS, IBM Watson Center Munich, Germany). Die Datenerhebung erfolgte durch persönlichen Kontakt mit Patienten oder Hausarzt. Kategoriale Variablen wurden in absoluten und relativen Häufigkeiten angegeben. Die Subgruppenanalyse erfolgte mittels dem exakten Test nach Fisher. Quantitative Werte wurden als Mediane mit Wertebereich angegeben, der Vergleich der Subgruppen erfolgte unter Verwendung des Mann-Whitney-U-Tests. *p*-Werte ≤ 0,05 galten als signifikant.

## Ergebnisse

### Patientenkollektiv

Die eingeschlossenen Patienten waren zum Operationszeitpunkt zwischen 11 und 59 Jahre alt. Der Median lag bei 22 Jahren. Der Großteil der Patienten war männlich (*n* = 32; 82 %). Die Beobachtungsdauer betrug 2 Jahre nach der Operation. Es wurden 21 (54 %) Patienten mit „akutem Pilonidalabszess“ und 18 (46 %) Patienten mit „chronischem Sinus pilonidalis“ eingeschlossen.

Von den Patienten mit „akutem Pilonidalabszess“ waren 12 Patienten (57 %) nicht voroperiert, 9 Patienten (43 %) waren ein- oder mehrfach am Pilonidalabszess voroperiert. Zwei Patienten (10 %) hatten einen Diabetes mellitus, 13 Patienten (62 %) einen fortgesetzten Nikotinabusus. Keiner der Patienten hatte eine periphere arterielle Verschlusskrankheit (pAVK). Nach Definition der WHO (World Health Organization) waren 8 Patienten (38 %) adipös mit einem Body-Mass-Index (BMI) > 30, der BMI lag im median bei 28 (18–39). 18 Patienten (86 %) waren männlich. Das Alter lag im Median bei 25 Jahren (14–59).

Von den Patienten mit „chronischem Sinus pilonidalis“ waren 14 (78 %) nicht voroperiert, 4 (22 %) waren ein- oder mehrfach an einem Pilonidalabszess voroperiert. Ein Patient (6 %) hatte zum Operationszeitpunkt einen behandelten Diabetes mellitus Typ 2. Acht Patienten (44 %) hatten zum Operationszeitpunkt einen fortgesetzten Nikotinabusus. Es wurde keine pAVK angegeben. Vier Patienten (22 %) hatten einen BMI von > 30, der BMI lag im Median bei 24 (19–38). Auch hier ist das männliche Geschlecht mit 14 Patienten (78 %) stärker vertreten. Das mediane Alter lag mit 18 Jahren (11–53) signifikant unter dem der akuten Gruppe (Tab. 1).

**Table 1:** 

	Akuter SP (*n* = 21)	Chronischer SP (*n* = 18)	Alle (*n* = 39)	*p*
*Geschlecht*				
Männlich	18 (86 %)	14 (78 %)	32 (82 %)	0,682
Weiblich	3 (14 %)	4 (22 %)	7 (18 %)	
*Medianes Alter in Jahren (Range)*	25 (14–59)	18 (11–53)	22 (11–59)	*0,030*
*Diabetes mellitus*	2 (10 %)	1 (6 %)	3 (8 %)	1,000
*Raucher*	13 (62 %)	8 (44 %)	21 (54 %)	0,343
*Adipositas*	8 (38 %)	4 (22 %)	12 (31 %)	0,322
*Rezidiveingriffe*	9 (43 %)	4 (22 %)	13 (33 %)	0,153

*SP* Sinus pilonidalis

### Perioperativer Behandlungsverlauf

In der Subgruppe des chronischen Plionidalsinus und der akut abszedierten Gruppe lag der stationäre Aufenthalt im Median bei 6 Tagen. Die Redondrainagen in beiden Gruppen wurden im Median nach 5 Tagen entfernt. Die antibiotische Therapie wurde in der akut abszedierten Gruppe durchschnittlich für 6 Tage appliziert. Drei Patienten (14 %) äußerten postoperative Schmerzen mit einem Schmerzskalenwert von 4 bis 5. In der Subgruppe des chronischen Pilonidalsinus erfolgte die Antibiotikatherapie im Median etwas länger über 7 Tage. Vermehrte postoperative Schmerzen wurden in dieser Gruppe nicht beobachtet.

### Therapieergebnisse

Postoperative Wundheilungsstörungen traten in beiden Gruppen in vergleichbarer Frequenz auf (10 % in der akuten gegenüber 17 % in der chronischen Subgruppe; *p* = 0,647). In der akut abszedierten Gruppe entwickelte ein Patient (5 %) ein Serom, das nach 3 Monaten operativ drainiert wurde. Ein Patient (5 %) entwickelte einen Abszess, der nach 9 Tagen postoperativ entstand und operativ entlastet und mittels offener Wundbehandlung zur Ausheilung gebracht wurde. In der chronischen Gruppe hatten 2 Patienten (11 %) eine oberflächliche Wundheilungsstörung, die konservativ zur Abheilung kam. Ein Patient (6 %) entwickelte einen lokalen Abszess, der 1 Monat nach der Limberg-Plastik auftrat und operativ entlastet wurde.

Während beim akuten Pilonidalabszess keine Rezidive auftraten, wurden in der chronischen Gruppe 2 Rezidive (11 %) erhoben. Der Unterschied war statistisch nicht signifikant (*p* = 0,206). Beide Abszesse konnten über eine operative Sanierung mit Einleitung einer offenen Wundtherapie zur Ausheilung gebracht werden.

Die Befragung der Patienten nach subjektiver Zufriedenheit mit dem Operationsergebnis war in beiden Gruppen vergleichbar. 15 Patienten (71 %) in der Gruppe des akuten Pilonidalabszesses und 12 Patienten (67 %) in der Gruppe des chronischen Sinus pilonidalis waren mit der Operation und ihrem Verlauf zufrieden und würden die Limberg-Plastik weiterempfehlen. Ein Patient (5 %) aus der akuten Gruppe vs. 2 Patienten (11 %) in der chronischen Gruppe enthielten sich (Tab. 2).

**Table 2:** 

	Akuter SP (*n* = 21)	Chronischer SP (*n* = 18)	Alle (*n* = 39)	*p*
Stationärer Aufenthalt (Tage)	6 (3–8)	6 (5–8)	6 (3–8)	1,000
Komplikationsrate Abszess/Wundheilungsstörung	2 (10 %)	3 (17 %)	5 (13 %)	0,647
Rezidiv	0 (0 %)	2 (11 %)	2 (5 %)	0,206
Subjektive Zufriedenheit	15 (71 %)	12 (67 %)	27 (69 %)	0,758

*SP* Sinus pilonidalis

## Diskussion

In dieser Studie zeigte sich eine Vergleichbarkeit der Ergebnisse des Limberg-Plastik-Verfahrens beim akuten und chronischen Sinus pilonidalis. Die Komplikations- und Rezidivraten waren insgesamt niedrig bei relativ hoher Patientenzufriedenheit in beiden Gruppen.

Während sich die deutsche Gesellschaft für Koloproktologie (DGK) mit anderen deutschen Gesellschaften in den S3-Leitlinien für eine Empfehlung der plastischen Techniken und Verschiebelappen aussprechen, werden in der internationalen Literatur die verschiedenen Vor- und Nachteile der Operationstechniken diskutiert und multizentrische Untersuchungen ausgearbeitet [1, 3, 6, 7, 9–11, 13–15, 18, 19]. So wurde eine Umfrage an allen Krankenhäusern und Privatkliniken in Dänemark durchgeführt, bei der die Entwicklung und Behandlung des Sinus pilonidalis untersucht wurde. In 11 Kliniken (40 %) wurden Lappenplastiken von nur 1 bis 2 erfahrenen Operateuren pro Klinik durchgeführt, im Bereitschaftsdienst wurde der Pilonidalsinus mittels Inzision oder Exzision und offener Wundheilung behandelt, was den geringen Anteil plastischer Eingriffe erklärt [20].

Auch in der Schweiz wurde in einer randomisierten kontrollierten multizentrischen Untersuchung über einen Zeitraum von 6,5 Jahren mit 102 Patienten ein Vergleich zwischen konventioneller Exzision mit offener Wundheilung und plastischem Verschluss nach Limberg vorgenommen. Für die konventionelle Exzision zeigte sich eine signifikant kürzere Operationszeit. Bezüglich der postoperativen Schmerzen und Patientenzufriedenheit konnte kein signifikanter Unterschied festgestellt werden. Es wurden mit 12 % signifikant weniger Wundheilungsstörungen bei der konventionellen Exzision als nach plastischem Verschluss nach Limberg (49 %) erhoben. In der Limberg-Gruppe fanden sich 13 % Rezidive [21]. Die Autoren schlussfolgern aus der hohen Komplikationsrate keine Überlegenheit der einzeitigen plastischen Deckung gegenüber der offenen Exzision. Wahrscheinlich sind diese hohen Komplikations- sowie Rezidivraten nach Limberg-Plastik auf eine geringe Expertise der Operateure zurückzuführen. In unserer Studie konnte mit 12 % eine deutlich geringere Komplikationsrate und mit 5 % eine geringere Rezidivrate für die einzeitige Limberg-Plastik demonstriert und die Ergebnisse der Schweizer Studie relativiert werden. In unserer Untersuchung wurde die Limberg-Plastik von erfahrenen Operateuren durchgeführt oder begleitet.

In einer systematischen weltweiten Analyse mit 6143 eingeschlossenen Studien wurde für die zweizeitige Limberg- und Dufourmentel-Plastik eine Rezidivrate von 0,6 % nach 12 Monaten und 1,8 % nach 24 Monaten erhoben [22]. Vergleichbar gute Ergebnisse mit 0 % konnten wir für die einzeitige Limberg-Plastik des akuten Pilonidalsinus herausarbeiten.

Beim akuten Pilonidalabszess wird von einem Großteil der Chirurgen eine einzeitige Operation mit offener Wundbehandlung durchgeführt. Dies ist wahrscheinlich dadurch begründet, dass Pilonidalabszesse keine planbaren Operationen sind und zeitliches Management notwendig ist, um den Operationsplan nicht zu verschieben. Andererseits kommen Patienten mit akutem Pilonidalabszess nicht selten spät abends oder nachts ins Krankenhaus. Das Operationsteam in der Nacht zu mobilisieren, ist möglicherweise aufwendiger, als einen Abszess in der Ambulanz unter Analgesie und Vereisung zu inzidieren. Damit würde das Operationsprogramm am Folgetag nicht verschoben werden müssen [23]. In eigenen, nicht publizierten Daten konnten wir eine geringe Anzahl begrenzter Pilonidalabszesse mittels kleiner Inzisionen und offener Wundheilung zur Abheilung bringen.

Minimal-invasive Techniken wie das Pit-Picking werden vermehrt bei begrenzten Befunden einfach und schnell durchgeführt, was für den Patienten mit weniger Schmerzen, kürzerem Krankenhausaufenthalt und kurzer Arbeitsausfallperiode verbunden ist [7, 24, 25].

Die internationale Studienlage ist weiterhin heterogen. Einerseits werden von Doll et al. in einer Studie von 2013 mit 583 Patienten eine geringe Langzeitrezidivrate für das zweizeitige Vorgehen als primäre Entlastung und definitiver chirurgischer Versorgung im entzündungsfreien Intervall beschrieben [26]. Dieses wird in einer Pilotstudie von Ardelt et al. 2015, der mit ähnlichen Ergebnissen ein zweizeitiges Vorgehen im Sinne einer Entlastung mit nachfolgendem definitivem Verschluss mittels Limberg-Lappenplastik empfiehlt, unterstützt [15]. Andererseits beschreiben Muzi et al. 2010 in einer randomisierten Studie eine höhere Rezidivrate bei der zweizeitigen Limberg-Plastik mit 3,84 % gegenüber dem Primärverschluss mit 0 % (nicht signifikant). In Bezug auf Schmerzfreiheit konnte in derselben Studie ein signifikanter Unterschied für den Primärverschluss erhoben werden [27]. Sevinç B et al. vergleichen die Exzision und spannungsfreien Primärverschluss mit der Karydakis- und Limberg-Plastik und erheben eine höhere Rezidivrate mit 6 % bei der Limberg- sowie Karydakis-Plastik gegenüber dem spannungsfreien Primärverschluss von 4 % nach Exzision [28].

Die Vorteile der Limberg-Plastik in Bezug auf die postoperative Rekonvaleszenz werden in anderen Studien deutlich. Akca et al. konnten bereits 2005 signifikant geringere postoperative Schmerzen, frühzeitige Mobilisation, einen kürzeren stationären Aufenthalt, kürzere berufliche Ausfallzeit sowie eine geringere Rezidivrate zugunsten der Limberg-Plastik im Vergleich zur Exzision und primärem Verschluss feststellen [1]. In einer prospektiv randomisierten kontrollierten Vergleichsstudie [13] mit 49 Patienten über einen Zeitraum von 3 Jahren wird ebenso ein Vorteil der Limberg-Plastik gegenüber der Exzision bezogen auf postoperative Schmerzen, stationären Aufenthalt, Zeit bis zur Heilung sowie Rezidivhäufigkeit dargestellt. In einer Pilotstudie wird die zweizeitige Vorgehensweise mit primärer Exzision und Limberg-Plastik sowohl beim akuten Pilonidalabszess als auch beim chronischen Pilonidalsinus wegen nicht aufgetretener Rezidive in einem Beobachtungszeitraum von 20 Monaten empfohlen. Allerdings traten 18,2 % Majorkomplikationen sowie 4,5 % Minorkomplikationen auf [15].

Die Exzision eines Sinus pilonidalis und Verschluss mittels Limberg-Plastik ist eine effektive chirurgische Methode, die mit einer geringen Komplikationsrate, geringer Rezidivrate und kurzer Hospitalisation einhergeht [8, 14]. Die Pilonidalsinusoperation und Rekonstruktion nach Limberg ist der simplen Pilonidalexzision und anschließendem Primärverschluss signifikant überlegen bezogen auf stationären Aufenthalt, Zeit der Wundheilung, Ausfallzeit der Arbeit, Wundinfektions- und Rezidivrate [29].

Bisher gilt die Annahme, einen abszedierenden Sinus pilonidalis zu inzidieren und zweizeitig eine definitive Versorgung mittels Limberg- oder Karydakis-Plastik durchzuführen [6, 7, 30].

Die Limberg-Plastik nach Exzision eines infizierten Sinus pilonidalis führt nicht nur zur frühen Wiederaufnahme der Arbeit, sondern auch zu einer im Vergleich zur sekundären Wundheilung elastischen und beweglicheren Narbe [2]. In der Literatur konnten nur wenige repräsentative Studien gefunden werden, die eine Exzision des abszedierenden Sinus pilonidalis mit definitiver Versorgung als Limberg-Plastik in primärer Sitzung beschreiben. Ciftci et al. vergleichen den primären Wundverschluss beim akuten Pilonidalabszess mit der Abszessdrainage und zeigen ein signifikant geringeres Risiko für Rezidive zugunsten des Primärverschlusses [10]. In einer weiteren Untersuchung konnten vergleichbare Ergebnisse der Limberg-Plastik einzeitig sowohl für den chronischen Pilonidalsinus als auch für den akuten Pilonidalabszess gefunden werden. Allerdings sind die Patientenzahlen für den akuten Pilonidalabszess gering [11]. Weitere Studien zum direkten Vergleich der offenen Exzision gegenüber dem primären Verschluss mittels (Limberg‑)Plastik beim akut abszedierten Befund wären nötig, um eine definitive Überlegenheit des einen oder anderen Operationsverfahrens in Bezug auf die postoperative Komplikationsrate, Rezidivrate und Rekonvaleszenz des Patienten zu ermöglichen.

Anhand der Ergebnisse unserer Studie stellt die definitive Versorgung des „akuten Pilonidalabszesses“ sowie des „chronischen Pilonidalsinus“ mittels Limberg-Plastik als einzeitige Operation eine valide Option zur herkömmlichen offenen Exzision dar.

Die perioperativen Ergebnisse und Rezidivraten unterscheiden sich beim „akut abszedierenden“ Sinus nicht signifikant von denen beim „chronischen Sinus pilonidalis“, insbesondere in Bezug auf die Entwicklung postoperativer Wundheilungsstörungen. Die Rezidivrate zeigt einen Trend zu einer geringeren Häufigkeit beim akuten gegenüber dem chronischen Befund.

Die Aussagekraft dieser Studie ist limitiert durch die geringe Patientenzahl und das Studiendesign. Es handelt sich hier nicht um einen randomisierten direkten Vergleich zweier Operationstechniken, sondern um eine Beobachtungsstudie einer Operationstechnik in zwei verschiedenen Patientenkollektiven. Die Komplikationsraten, die von uns festgestellt wurden, liegen deutlich unter den in der Literatur beschriebenen, dies mag möglicherweise in der chirurgischen Expertise, möglicherweise aber auch in dem relativ jungen Patientengut mit geringer Komorbidität begründet sein.

## Fazit für die Praxis

- In dieser Studie zeigten sich die postoperativen Ergebnisse der einzeitigen operativen Versorgung beim akuten und chronischen Sinus pilonidalis vergleichbar.
- Die bisherigen Leitlinien empfehlen die Exzision oder das zweizeitige Vorgehen mittels Lappenplastik für den chronischen Sinus pilonidalis. Beim akut abszedierten Befund ist nach mehrheitlicher Meinung sowie Leitlinienempfehlung ebenfalls ein zweizeitiges Vorgehen vorzuziehen.
- Unsere Daten zeigen nun eine Gleichwertigkeit des einzeitigen Vorgehens bei akuten und chronischen Befunden und vergleichbare Ergebnisse zu den Literaturergebnissen des zweizeitigen Vorgehens mit plastischer Rekonstruktion.
- In Anlehnung an unsere Erhebung sollten sich daher weitere Untersuchungen anschließen, um zu eruieren, ob ein einzeitiges Vorgehen dem zweizeitigen auch bei akuten Befunden gleichzusetzen oder sogar überlegen ist.
- Ein erfolgreiches einzeitiges operatives Vorgehen erspart dem Patienten einen erneuten operativen Eingriff sowie weiteren Schmerzen ausgesetzt zu sein und verkürzt potenziell die beruflichen Ausfallzeiten und verringert somit die sozioökonomische Belastung.
